# A meta-analysis of *caspase-8* -652 6N del polymorphism and digestive tract cancer risk

**DOI:** 10.7555/JBR.32.20160030

**Published:** 2017-11-28

**Authors:** Haina Du, Guoxin Song, Mingzhi Fang, Yongqian Shu, Xin Zhao, Lingjun Zhu

**Affiliations:** 1. Department of Oncology, the Third Affiliated Hospital of Nanjing University of T.C.M, Nanjing, Jiangsu 210000, China; 2. Departments of Pathology, the First Affiliated Hospital of Nanjing Medical University, Nanjing, Jiangsu 210009, China; 3. Departments of Oncology, the First Affiliated Hospital of Nanjing Medical University, Nanjing, Jiangsu 210009, China; 4. Departments of Pneumology, the First Affiliated Hospital of Nanjing Medical University, Nanjing, Jiangsu 210009, China.

**Keywords:** caspases-8, polymorphism, digestive tract cancer risk

## Abstract

Caspase-8 (CASP8) is one key regulator of apoptosis of T lymphocytes and is encoded by the *CASP8* gene. It has been reported that the six-nucleotide deletion polymorphism (-652 6N del) of the *CASP8* gene had effect on some cancer risk. Few studies explored the association between *CASP8* gene polymorphism and digestive tract cancer risk. To evaluate the association between the *CASP8* -652 6N del polymorphism and the risk of digestive tract cancer, we conducted this meta-analysis. We found that *CASP8*-652 6N del polymorphism was associated with a significantly reduced risk of digestive tract cancer in the co-dominant model (del/del* vs. *ins/ins: OR= 0.82, 95%CI= 0.72–0.95; del/ins* vs. *ins/ins: OR= 0.92, 95%CI= 0.87–0.97; dominant model (del/ins+ del/del* vs. *ins/ins: OR= 0.91, 95%CI= 0.87–0.96, recessive model: del/del* vs. *del/ins+ ins/ins: OR= 0.85, 95%CI= 0.75–0.97). In the stratified analysis by cancer types, we found that all genetic models had protective effect on gastric cancer. Similar results were observed for colorectal cancer under heterozygote comparison and dominant model, but not under homozygote comparison or recessive model. In addition, a significantly decreased risk was found on esophageal cancer for most genetic models, except heterozygote comparison. When stratified by ethnicity and source of control, an evidently decreased risk was identified in the Asian populations and population-based studies. In conclusion, there exists an association between the *CASP8* -652 6N del polymorphism and reduced digestive cancer risk, especially among Asians and population-based studies.

## Introduction

The activity of many genes influences a cell's likelihood of activating its apoptotic program^[1]^, which is mainly regulated by caspases, a family of cysteine proteases, that play a crucial role in the development and progression of cancer^[2]^. Several meta-analyses have confirmed that the gene variants of caspases-2, 5, 7, 8, 9 and 10 disturbed the apoptotic mechanism and thus affected the risk of some cancers^[3^–^10]^.

Caspase-8 (CASP8) is a key regulator of apoptosis of T lymphocytes and is encoded by the *CASP8* gene^[8]^. The *CASP8* (*MACH*, *FLICE*, *Mch5*) gene contains at least 11 exons spanning ~30 kb on human chromosome band 2q33–34^[11]^ and encodes 479 amino acids, including prodomain and caspase domain. There are 353 single-nucleotide polymorphisms (SNP) of the *CASP8* reported in the dbSNP database^[12]^. It has been reported that polymorphic variation in *CASP8* influences cancer risk, such as the variant D302H (rs1045485), the 652 6N insertion/deletion (ins/del) promoter variant (rs3834129), and the IVS12-19G/A (rs3769818). Previous meta-analysis suggested *CASP8* -652 6N promoter polymorphism is associated with reduced renal cell carcinoma risk^[5]^ and breast cancer risk^[10]^. However, there was no evidence on the association between the -652N ins/del and digestive tract cancer due to limited publications. Digestive tract cancers represent a homogeneous group of malignancies. Given the amount of data now available on the association between *CASP8* -652 6N promoter polymorphism and digestive tract cancer susceptibility, we performed the meta-analysis based on published case-control studies.

## Materials and methods

### Search strategy and inclusion criteria

We carried out a search in PubMed and Embase databases with the following keywords: ('CASP8' or 'caspase-8', or 'rs3834129', 'cancer' or 'neoplasm' or 'carcinoma' or 'tumor' and 'polymorphism' or 'single nucleotide polymorphisms' or 'SNPs' (last search was updated on Dec 31, 2015). The titles and abstracts of all eligible studies were examined carefully, and the bibliographies of the selected papers were also checked manually. If the same patient population was included in multiple publications, only the most recent or complete study was used in this meta-analysis.

Selected studies in our meta-analysis had to meet all the following criteria: (a) only case-control or cohort studies; (b) evaluation of the *CASP8* -652 6N promoter polymorphism and digestive system cancer risk; (c) data available on genotype frequency or the value of the OR.

### Data extraction

For each study, the following information was extracted by two evaluators: first author's name, year of publication, country, ethnicity, source of control, sample size and genotyping results of cases and controls (*Table 1*). Data were independently extracted by two investigators. If consensus was not reached by discussion, the third investigator was rereading.

### Statistical analysis

Our meta-analysis assessed the overall association between *CASP8* -652 6N promoter polymorphism and digestive system cancer risk. The pooled ORs were performed for homozygote comparison (del/del* vs. *ins/ins), heterozygote comparison (del/ins* vs. *ins/ins), dominant model (del/ins+ del/del* vs. *ins/ins), and recessive model (del/del* vs. *del/ins+ ins/ins), respectively. *P*<0.05 was considered as statistically significant. Subgroup analyses were carried out by the cancer types and ethnicity, respectively. We used either the fixed (Mantel-Haenszel) or random (DerSimonian-Laird) effects models to get pooled ORs. If the *P* was more than 0.05 for the Q-test, the heterogeneity was considered significant. When there was no heterogeneity among studies, the pooled OR estimate of each study was calculated by the fixed-effects model^[13]^. Otherwise, the random-effects model was used^[14]^. The Begg's test and the Egger's test were used to test possible publication bias in this meta-analysis. Hardy–Weinberg equilibrium (HWE) in the controls was evaluated using the chi-square test. Meta-analyses were performed using Stata 11.0 software.

## Results

### Study characteristics

**Fig.1:**
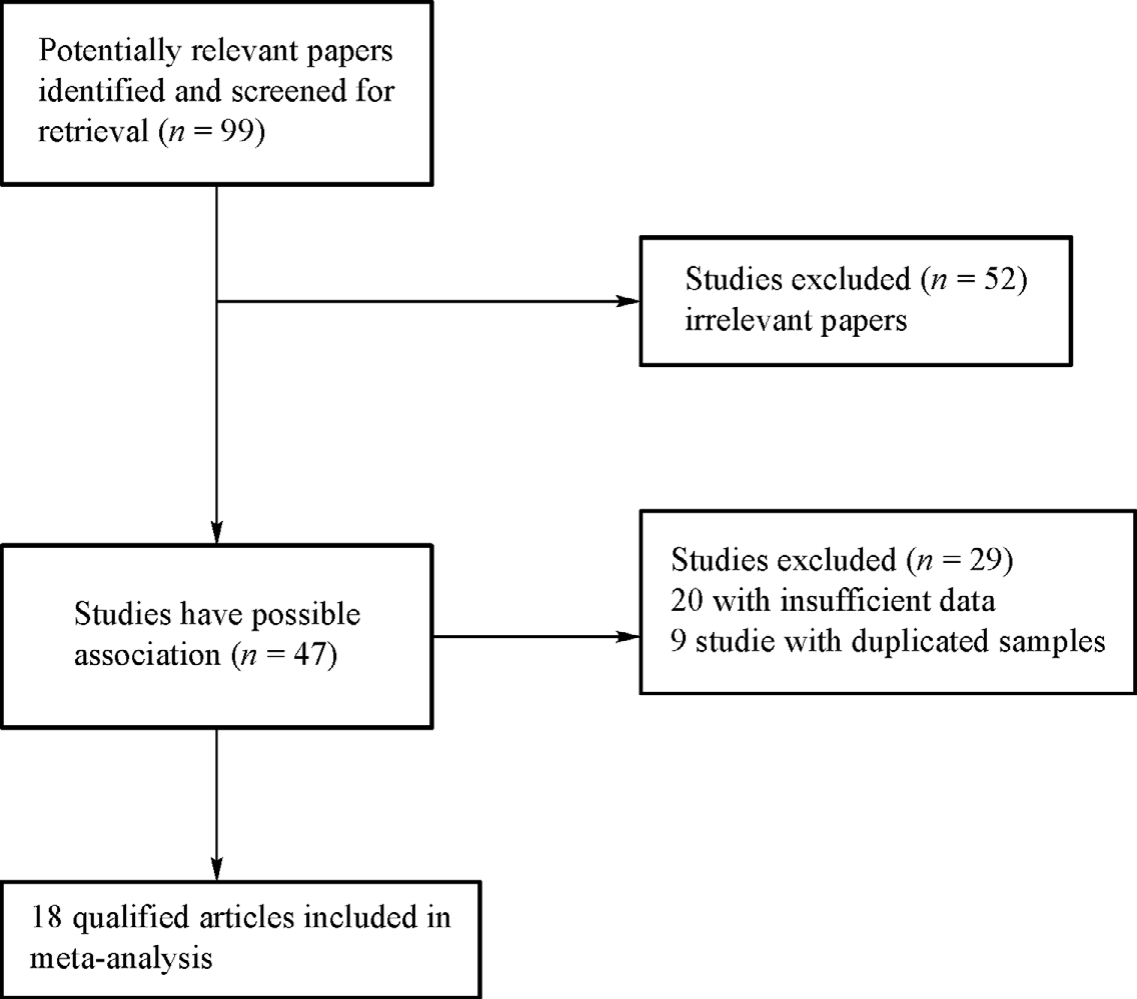
Flow diagram of study identification.

As shown in *Table 1*, 18 separate studies including a total of 15,086 patients and 16,374 controls were finally retrieved. Among them, there were 3 esophageal cancer studies^[12^,^15^–^16]^, 3 gastric cancers^[12^,^15^,^17]^ and 12 colorectal cancers (including studies)^[15^,^18^–^23]^ (*Fig. 1*). In addition, there were 9 studies in Asians populations, 9 in Caucasians populations and 13 were population-based studies, and 5 hospital-based studies. The distribution of genotypes in the controls was consistent with HWE, except for only one study ^[16]^.

**Tab.1:** Characteristics of studies included in the meta-analysis

Study	Year	Country	Ethnicity	Cancer	Design	Number	Genetypes (case/control)			*P* for HWE
						Case/control	6N ins/ins	6N del/ins	6N del/del	
Sun	2007	China	Asian	Esophageal	PB	1,018/937	652/543	328/338	38/56	0.724
Umar	2011	Indian	Asian	Esophageal	PB	259/259	139/138	103/93	17/28	0.046
Mailk	2011	Indian	Asian	Esophageal	PB	135/195	68/96	59/75	8/24	0.126
Sun	2007	China	Asian	Gastric	PB	420/410	262/233	142/152	16/25	0.974
Liamarkopoulos	2011	Greece	Caucasian	Gastric	HB	88/480	35/120	42/254	11/106	0.194
Mailk	2011	Indian	Asian	Gastric	PB	108/195	59/96	44/75	5/24	0.126
Sun	2007	China	Asian	Colorectal	PB	918/890	605/528	280304	33/58	0.116
Pittman	2008	UK	Caucasian	Colorectal	PB	3,879/2,777	995/8	1,897/1,872	987/897	0.170
Liu	2010	China	Asian	Colorectal	PB	370/1202	233/892	116/278	21/32	0.538
Theodoropoulos	2011	Greece	Caucasian	Colorectal	HB	402/480	103/120	201/254	98/106	0.194
Xiao	2013	China	Asian	Colorectal	HB	305/342	187/212	107/115	11/15	0.904
Wu	2013	China	Asian	Colorectal	HB	451/631	284/358	152/244	15/29	0.118
Pardini	2014	Spanish	Caucasian	Colorectal	PB	1,978/1,647	500/425	996/802	482/420	0.289
*Ibid*		Italian	Caucasian	Colorectal	PB	617/2,551	195/783	285/1,230	137/538	0.177
*Ibid*		USA	Caucasian	Colorectal	PB	1,010/1,580	237/383	514/794	259/403	0.835
*Ibid*		English	Caucasian	Colorectal	PB	1,576/767	410/165	825/393	341/209	0.435
*Ibid*		Czech	Caucasian	Colorectal	HB	967/672	239/169	479/326	249/177	0.442
*Ibid*		Dutch	Caucasian	Colorectal	PB	585/359	169/106	282/177	134/76	0.894

PB: population-based; HB: hospital-based.

### Meta-analysis results

**Fig.2:**
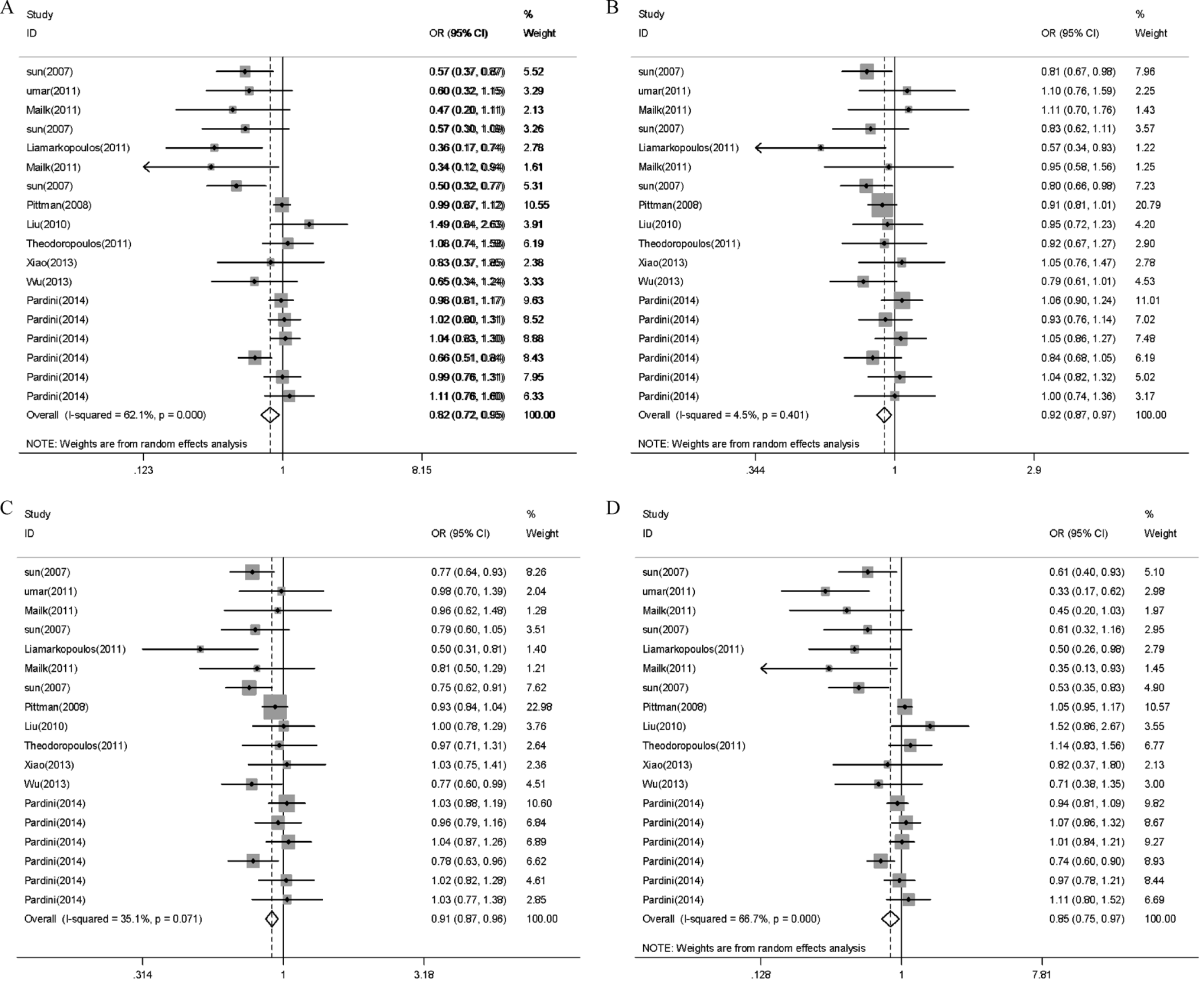
Odds ratios (ORs) for associations between *CASP8* 652 6N ins/del polymorphisms and digestive system cancer risk based on all models. A: del/del *vs.* ins/ins, B: del/ins vs. ins/ins, C: del/del + del/ins vs. ins/ins; D: del/del vs. del/ins + ins/ins.

Overall, a significant reduced risk of digestive tract cancer was associated with the *CASP8*-652 6N del polymorphism for the co-dominant model (del/del* vs. *ins/ins: OR= 0.82, 95%CI= 0.72–0.95, *Fig. 2A*; del/ins* vs. *ins/ins: OR= 0.92, 95%CI= 0.87–0.97, *Fig. 2B*); dominant model (del/ins+ del/del* vs. *ins/ins: OR= 0.91, 95%CI= 0.87–0.96, *Fig. 2C*), recessive model (del/del* vs. *del/ins+ ins/ins: OR= 0.85, 95%CI= 0.75–0.97, *Fig. 2D*).

In the stratified analysis by cancer types, we found all genetic models had protective effect on gastric cancer: del/del* vs. *ins/ins: OR= 0.43, 95%CI= 0.28–0.67; del/ins* vs. *ins/ins: 0.79 (0.63–0.99); del/ins+ del/del* vs. *ins/ins: 0.73 (0.59–0.95); del/del* vs. *del/ins+ ins/ins: 0.50 (0.33–0.77). Similar results were observed for colorectal cancer under heterozygote comparison (OR= 0.94, 95%CI= 0.88–0.99, *Supplementary Fig. 1A*, available online) and dominant model (OR= 0.93, 95%CI= 0.88–0.99, *Supplementary Fig. 1B*, available online), but not under homozygote comparison or recessive model. In addition, a significant decreased risk was found on esophageal cancer for the mostly genetic models: del/del* vs. *ins/ins: 0.56 (0.40–0.78); del/ins+ del/del* vs. *ins/ins: 0.83 (0.71–0.97); del/del* vs. *del/ins+ ins/ins: 0.57 (0.41–0.79), except the heterozygote comparison.

When stratified by ethnicity, two distinct results appeared in Asian populations and European populations. An evidently decreased risk for Asian populations in any genetic models (del/del* vs. *ins/ins: OR= 0.62, 95%CI= 0.51–0.76, *Supplementary Fig. 2A*, available online; del/ins* vs. *ins/ins: OR= 0.87, 95%CI= 0.79–0.95, *Supplementary Fig. 2B*, available online; del/ins+ del/del* vs. *ins/ins: OR= 0.83, 95%CI= 0.76–0.91, *Supplementary Fig. 2C*, available online; del/del* vs. *del/ins+ ins/ins: OR= 0.61, 95%CI= 0.46–0,83, *Supplementary Fig. 2D*, available online). In stratified analysis by source of control, we found significant reduced CRC risk in population-based studies (del/del* vs. *ins/ins: OR= 0.82, 95%CI= 0.70–0.96; del/ins* vs. *ins/ins: OR= 0.92, 95%CI= 0.87–0.98; del/ins+ del/del* vs. *ins/ins: OR= 0.91, 95%CI= 0.86–0.96; del/del* vs. *del/ins+ ins/ins: OR= 0.83, 95%CI= 0.71–0.97), but not in hospital-based studies (figure not shown).

### Publication bias

**Fig.3:**
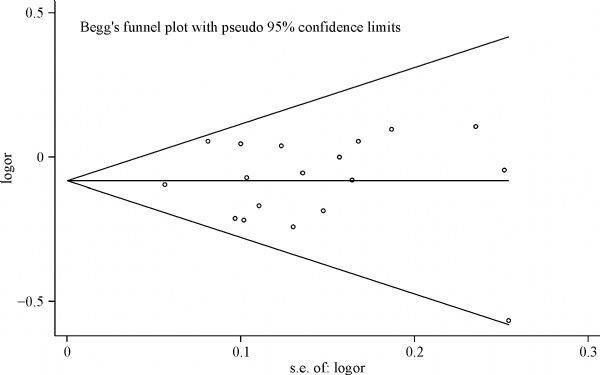
Funnel plot analysis to detect publication bias for the *CASP8*-652 6N del polymorphisms (del/ins *vs. *ins/ins). Each point represents an individual study for the indicated association

Begg's funnel plot and egger's test were used to assess the publication bias. As shown in *Fig. 3*, the shape of the funnel plot for the heterozygous model did not show any obvious asymmetry, which suggested no publication bias existing in all comparison models. And the P values were 0.914, 95%CI were −1.461–1.317 in Egger's test, insinuating no publication bias (there were no publication bias in other genetic models, data not shown).

### Sensitivity analysis

**Fig.4:**
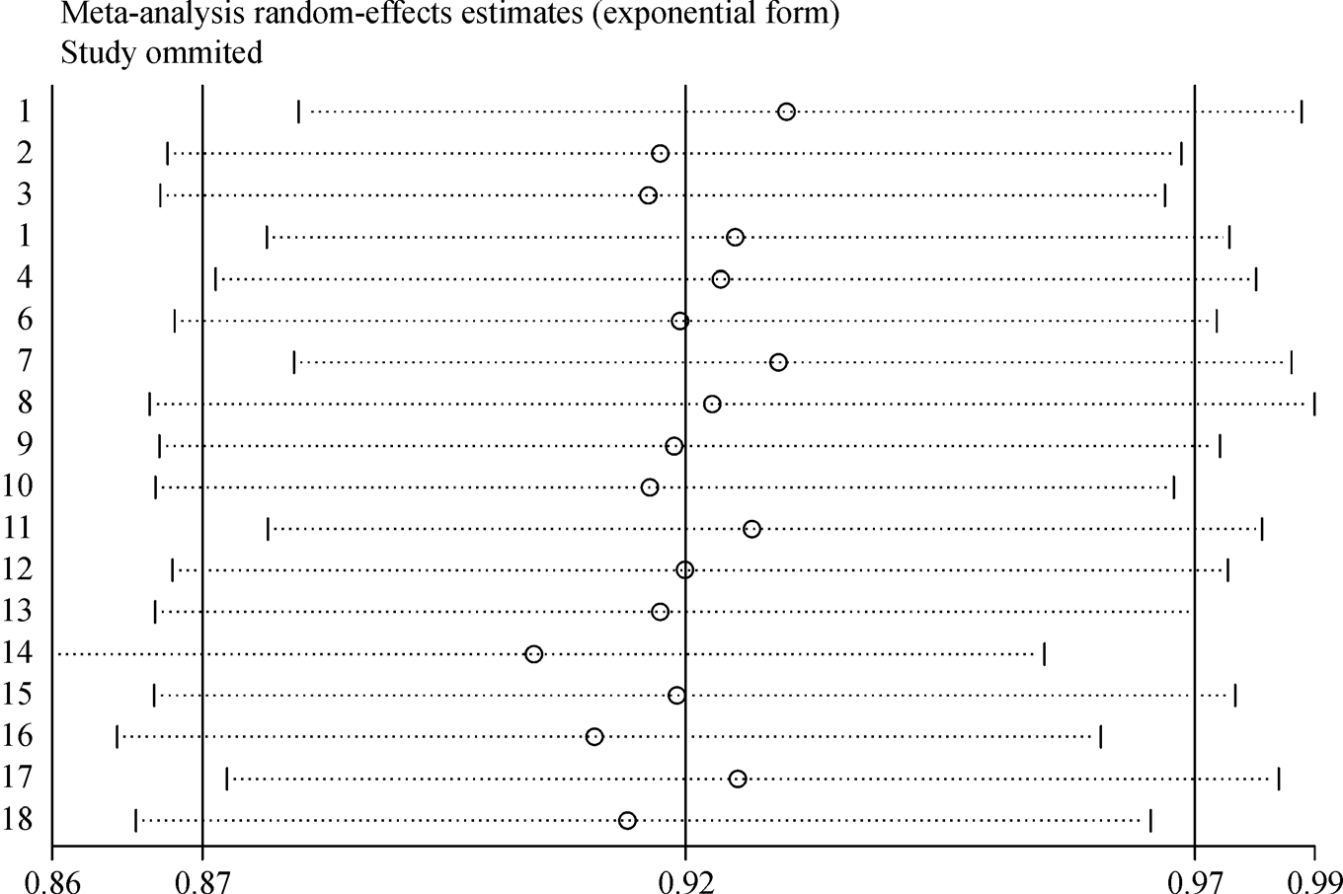
Influence analysis of the summary odds ratio coefficients on the association between *CASP8*-652 6N del polymorphisms (del/ins *vs.* ins/ins) with digestive tract cancer risk. Results were computed by omitting each study (left column) in turn. Bars, 95% confidence interval.

Sensitivity analysis was conducted repeatedly when the special studies had been left out at a time. For example, after excluding the study deviated from HWE^[16]^, the overall result did not been influenced significantly (heterozygous model: OR= 0.84, 95% CI: 0.73–0.96). The sensitivity analysis results revealed that no individual studies significantly affected the pooled ORs under heterozygous model of *CASP8* -652 6N polymorphism (*Fig. 4*), suggesting that our results are stable.

### Heterogeneity analysis

There was potential heterogeneity among studies in overall analysis and some subgroup analysis under homozygous and recessive model (*Table 2*). To explore the source of heterogeneity, we conducted a subgroup analysis by cancer types, ethnicity and the source of control. As a result, there presented little or no heterogeneity across studies. Furthermore, we found that colorectal cancer, Caucasian and population-based studies contributed to substantial heterogeneity.

**Tab.2:** Associations between *CASP8*-652 6N ins/del genotype and digestive tract cancer risk

Stratification	Total	Colorectal cancer	Gastric cancer
del/del *vs*. ins/ins			
OR (95%CI)	0.82(0.72–0.95)	0.93(0.82–1.05)	0.43(0.28–0.67)
*P*	0.01	0.247	<0.001
*P*_*h*_	<0.001	0.018	0.554
del/ins *vs*. ins/ins			
OR (95%CI)	0.92(0.87–0.97)	0.94(0.88–0.99)	0.79(0.63–0.99)
*P*	0.004	0.025	0.043
*P*_*h*_	0.401	0.529	0.304
del/del *vs*. del/ins+ins/ins			
OR (95%CI)	0.85(0.75–0.97)	0.96(0.87–1.07)	0.73(0.59–0.95)
*P*	0.013	0.444	0.004
*P*_*h*_	<0.001	0.019	0.241
del/del+del/ins *vs.* ins/ins			
OR (95%CI)	0.91(0.87–0.96)	0.93(0.88–0.99)	0.50(0.33–0.77)
*P*	<0.001	0.016	0.001
*P*_*h*_	0.071	0.190	0.641

*P*: *P* value of the comparison of ORs; *P*_*h *_: *P* value of the Q-test for heterogeneity test.

## Discussion

Apoptosis plays critical roles in a wide variety of physiologic processes during fetal development and in adult tissues^[24]^. Defects in apoptotic cell death regulation contribute to many diseases, such as some cancers^[24]^. Caspase 8 is a central regulator of apoptosis or programmed cell death.* Caspase-8* gene mutation can influence the rate of apoptosis, and thus affect cancer risk. The most widely reported mutation in *CASP8* gene is the six-nucleotide deletion polymorphism (-652 6N del). The-652 6N del variant involves a six-nucleotide deletion in the promoter region of the *CASP8* gene^[15]^. The deletion abolishes an Sp1 binding site and is associated with decreased RNA expression in lymphocytes and lower CASP8 activity and activation-induced cell death of T lymphocytes^[25]^.

It is known that apoptosis plays an important role in cancer growth, progression, and drug resistance^[26]^. Previous studies found that genetic mutations of caspases 8 in tumor cells reduce the cell sensitivity to apoptosis, which may be related to the occurrence and development of tumors. Our meta-analysis based on 18 case-control studies suggested that the *CASP8* -652 6N del polymorphism is associated with decreased risk of digestive tract cancer, which was similar with the results of Yin *et al.*^[27]^ and Zhang *et al.*^[28]^. However, there was potential heterogeneity under the four genetic models in digestive system cancer. Therefore, we also carried out the stratification analysis by ethnicity, cancer type and source of control. In stratification by cancer type, we also found the reduced cancer risk remained for subgroups of colorectal cancer, gastric cancer and esophageal cancer. What is more, similar results were found in Asians and population-based studies. Peng *et al.*^[29]^ reached a similar conclusion that *CASP8 *-652 6N ins/del polymorphism may play a protective role in colorectal cancer development. But their sample size was relatively small, not having enough statistical power to explore the real association. Our study with a large sample size provides additional evidence of the association between *CASP8* -652 6N ins/del polymorphism and colorectal cancer risk.

There was a significant reduced association between the-652 6N del polymorphism and digestive tract cancer risk among Asian populations, but not in Caucasians. One explanation may be genetic differences and diverse living environments between Asian and Caucasian populations. On the other hand, the Asian population are relatively homogeneous, who mainly come from Han Chinese. However, Caucasian population consisted of people from various countries with diverse living environments. Difference between Asians and Caucasians may explain the distinct findings. In addition, gene-gene and gene-environmental interactions may lead to the result bias. Among the included studies, 13 were population-based samples and 5 were hospital-based samples, respectively. Borderline significantly decreased risks were found for population-based studies. One explanation may be the hospital-based studies have some biases because such controls may be a sample of ill-defined reference population, particularly when the genotypes under investigation were associated with the disease conditions^[30]^. On the other hand, the insufficient hospital-based samples tended to have underestimated cancer risks.

Some limitations of this meta-analysis should be addressed. First, in the subgroup analyses of cancer type, the number of esophageal cancer and gastric cancer were relatively small, not having sufficient statistical power to explore the real association. Second, our results were based on unadjusted estimates, because not all published studies presented adjusted ORs. More precise analysis should be carried out, which would allow for the adjustment by other variance, such as sex, age, smoking status, drinking status, obesity and other lifestyles. Thirdly, the experiment method for each study was without restrictions, which affected the accuracy of the results to a certain extent.

In conclusion, our meta-analysis supports the association between the *CASP8*-652 6N del polymorphisms and reduced digestive tract cancer risks, especially in Asians. Future studies with large sample sizes are required to confirm current findings.
